# Supplementary material for: An ISO-certified genomics workflow for identification and surveillance of antimicrobial resistance
Source: Nat Commun. 2023 Jan 4;14:60. doi: 10.1038/s41467-022-35713-4 (PMC9813266; doi:10.1038/s41467-022-35713-4)
Supplement: Supplementary file 1 — Supplementary Information [file 41467_2022_35713_MOESM1_ESM.pdf]

An ISO-certified genomics workflow for identification and surveillance of antimicrobial resistance

Norelle L. Sherry, Kristy Horan, Susan A. Ballard, Anders Gonçalves da Silva, Claire L. Gorrie, Mark B. Schultz, Kerrie Stevens, Mary Valcanis, Michelle Sait, Timothy P. Stinear, Benjamin P. Howden and Torsten Seemann

Supplementary Information: Contents

SUPPLEMENTARY FIGURES

Supplementary Figure 1. Reporting logic – AMR gene reporting *Page 3*

Supplementary Figure 2. Reporting logic – Inferred antibiogram reporting *Page 4*

Supplementary Figure 3. Species used in validation of carbapenemase and ESBL detection *Page 5*

Supplementary Figure 4. Gene targets included in validation of carbapenemase and ESBL detection *Page 6*

Supplementary Figure 5. *Enterococcus* and *Staphylococcus* species included in validation of vancomycin resistance gene detection *Page 7*

Supplementary Figure 6. Gene targets included in validation of vancomycin detection *Page 7*

Supplementary Figure 7. Resistance alleles used in validation of allele calling *Page 8*

Supplementary Figure 8. Genera and species included in validation of resistance allele calling *Page 9*

Supplementary Figure 9. Genera and species included in validation of resistance allele calling from synthetic dataset *Page 10*

Supplementary Figure 10. Drug classes with represented in synthetic dataset for the validation of resistance allele calling *Page 11*

SUPPLEMENTARY TABLES

Supplementary Table 1. Performance of *abritAMR* using different genome assembly tools *Page 12*

Supplementary Table 2. Validation results for inferred phenotype in *Salmonella* spp. *Page 13*

Supplementary Table 3. Resistance classes and gene targets determined using synthetic reads *Page 14*

Supplementary Table 4. Steps and logic for *abritAMR* Classification Database updates *Page 17*

Supplementary Table 5. Gene targets and PCR primers for carbapenemase and ESBL detection panel *Page 20*

Supplementary Table 6. Isolates and sequence runs used to determine precision *Page 21*

SUPPLEMENTARY METHODS

Summary of accredited genomic sequencing workflow, MDU PHL *Page 22*

Overview of accredited genomic sequencing workflow

Detailed steps – inputs, controls, pick-off, DNA extraction & normalisation, library preparation and quality control, library pooling, normalisation and sequencing

Quality control – sequence run QC, PhiX control, QC of reads (bioinformatics)

Supplementary References *Page 21*

Supplementary Figure 1. Reporting logic – AMR gene reporting

This figure details the logic used for the final step of *abritAMR*, to determine whether each result is placed in to 'Reportable' or 'Non-reportable' fields of the Final AMR gene Report output (for clinical microbiology reports).

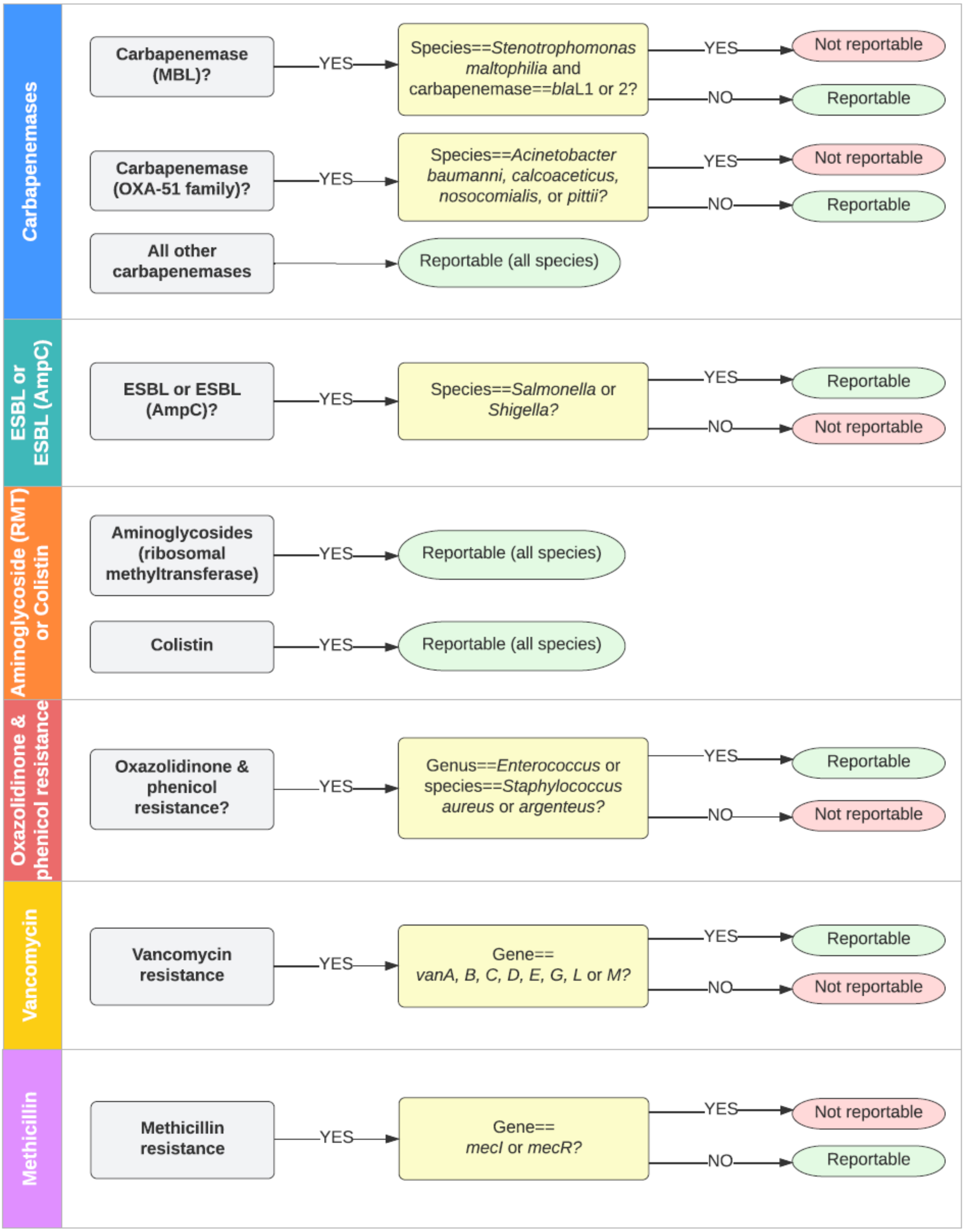

Supplementary Figure 2. Reporting logic – Inferred antibiogram reporting

IF ANY OF THE FOLLOWING DETECTED >> RESISTANT
OTHERWISE SUSCEPTIBLE

Antibiotic	Rule
Ampicillin	**Any gene from enhanced subclasses** ==Beta-lactamase (narrow spectrum) ==Beta-lactamase (not ESBL or carbapenemase) ==Cefotaxime (ESBL) ==Cefotaxime (AmpC type), or contains "Carbapenem"
Cefotaxime (ESBL)	**Any gene from enhanced subclasses** ==ESBL, or contains "Carbapenem"
Cefotaxime (AmpC type)	**Any gene from enhanced subclasses** ==ESBL (AmpC type), or contains "Carbapenem"
Meropenem	**Any gene from enhanced subclasses** contains "Carbapenemase" except "Carbapenemase (KPC variant)"
Tetracycline	**Any gene from enhanced subclass** contains "Tetracycline"
Azithromycin	**Any gene or mutation from enhanced subclass:** contains "Azithromycin"

IF ANY OF THE FOLLOWING DETECTED>> RESISTANT
OTHERWISE SUSCEPTIBLE

Antibiotic	Rule
Trimethoprim	**Any gene from enhanced subclass** ==Trimethoprim
Sulfathiazole	**Any gene from enhanced subclass** ==Sulfonamide
Trimethoprim-Sulfamethoxazole	**Any gene from enhanced subclasses** ==Trimethoprim, or ==Sulfonamide
Gentamicin	**Any gene from enhanced subclasses** contains "Gentamicin" or contains "Aminoglycoside"
Kanamycin	**Any gene from enhanced subclass** contains "Kanamycin"
Streptomycin	**Any gene from enhanced subclass** contains "Streptomycin"
Chloramphenicol	**Any gene from enhanced subclass** contains "Phenicol"

SPECIAL CASES

Ciprofloxacin

AMR genes or mutations from enhanced subclasses contain "Ciprofloxacin" or "Quinolone"

No AMR genes or mutations >> **Susceptible**

One AMR gene or mutation >> **Intermediate**

Two or more AMR genes or mutations >>**Resistant**

REPORTING OF AMR GENE CLASSES ONLY (NO INFERRED ANTIBIOGRAM)

Aminoglycosides (ribosomal methyltransferases)	Reportable mechanism
Colistin	Mobile colistin resistance (*mcr*) genes<br>Reportable mechanism
Other	AMR genes not reported under any other category; recorded in LIMS, not included in routine reports

Supplementary Figure 3: Species used in validation of carbapenemase and ESBL detection

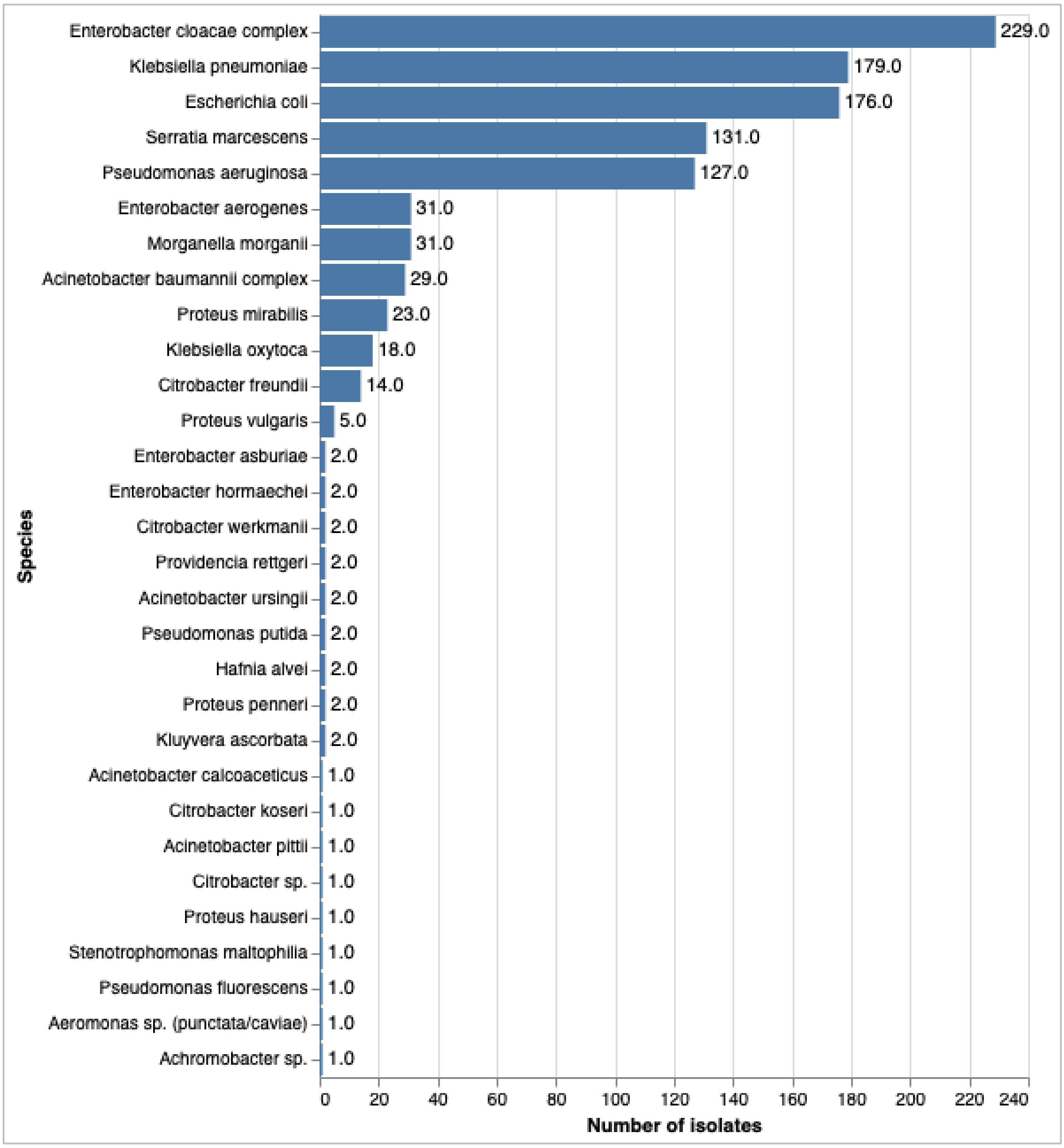

Source data are provided as a Source Data file.

Supplementary Figure 4: Gene targets included in validation of carbapenemase and ESBL detection

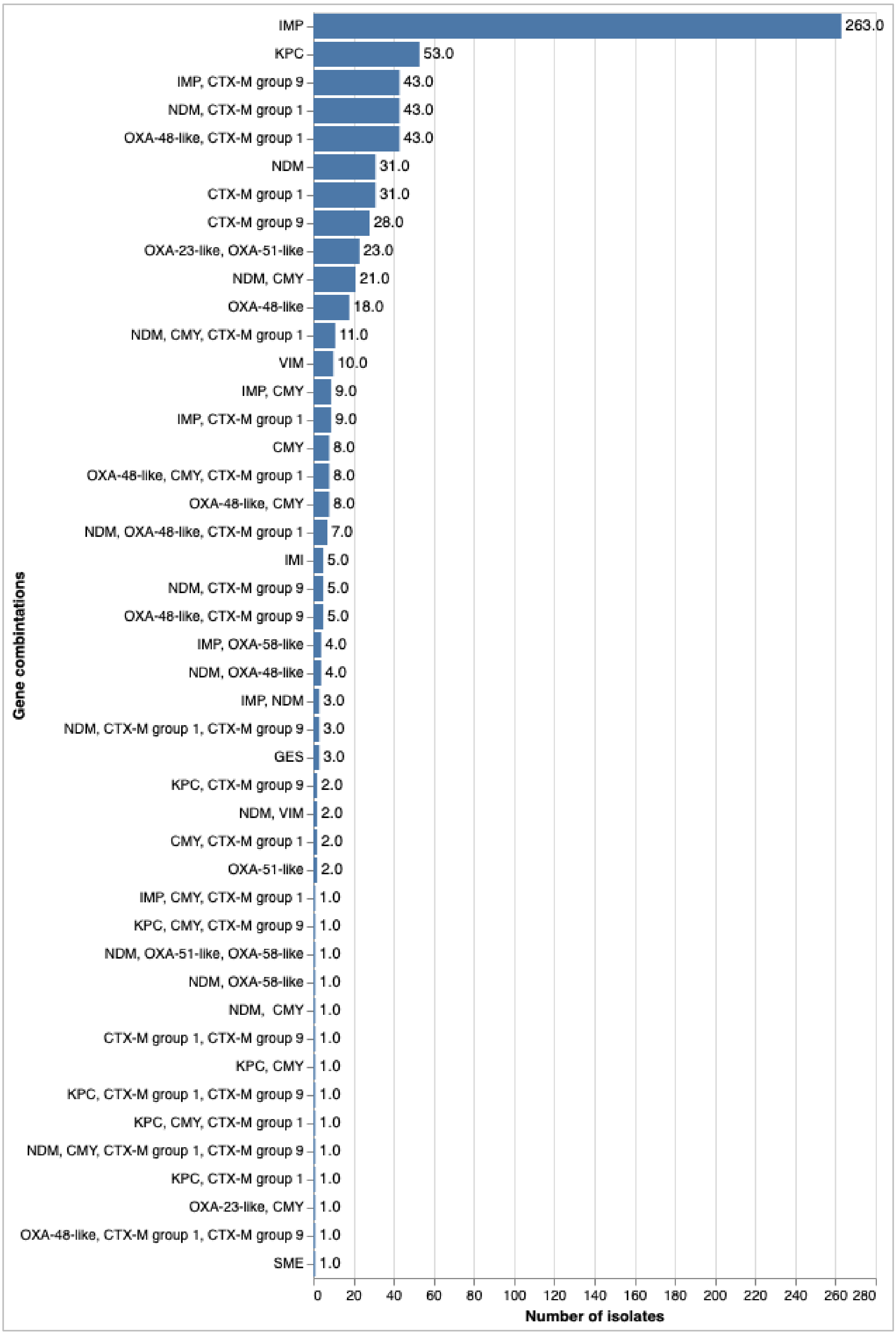

Source data are provided as a Source Data file.

Supplementary Figure 5: *Enterococcus* and *Staphylococcus* species included in validation of vancomycin resistance gene detection

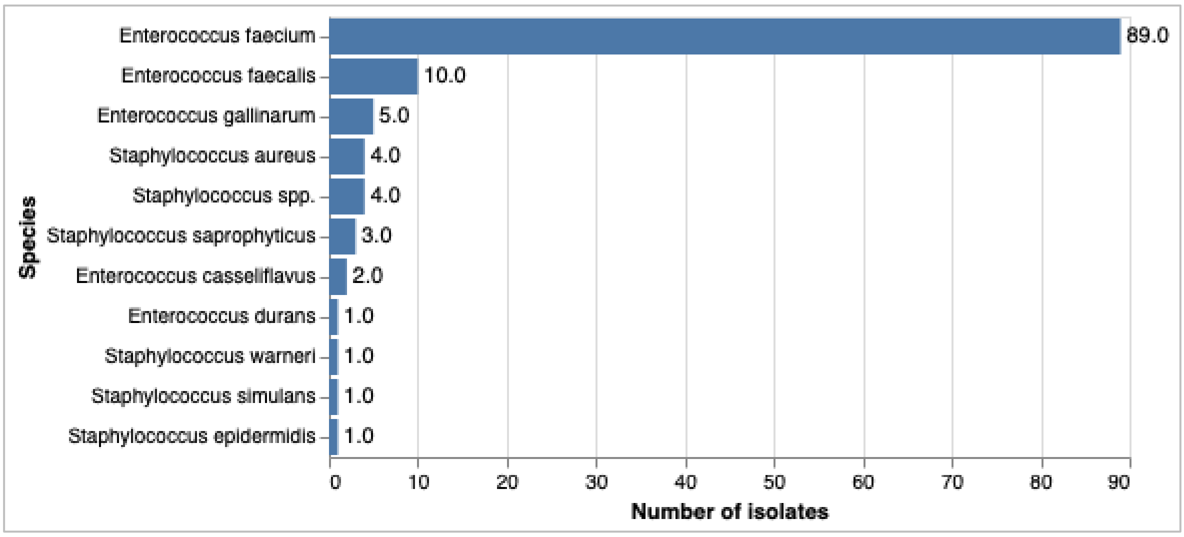

Source data are provided as a Source Data file.

Supplementary Figure 6: Gene targets included in validation of vancomycin detection

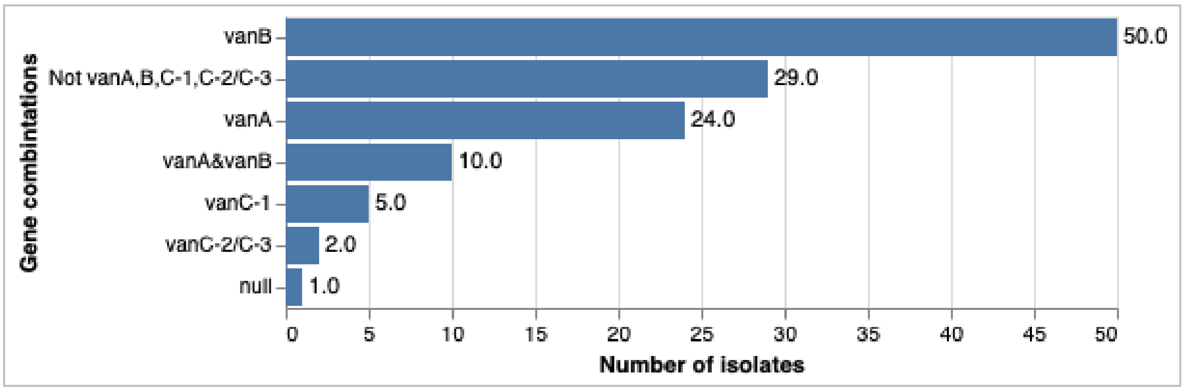

Source data are provided as a Source Data file.

Supplementary Figure 7. Resistance alleles used in validation of allele calling

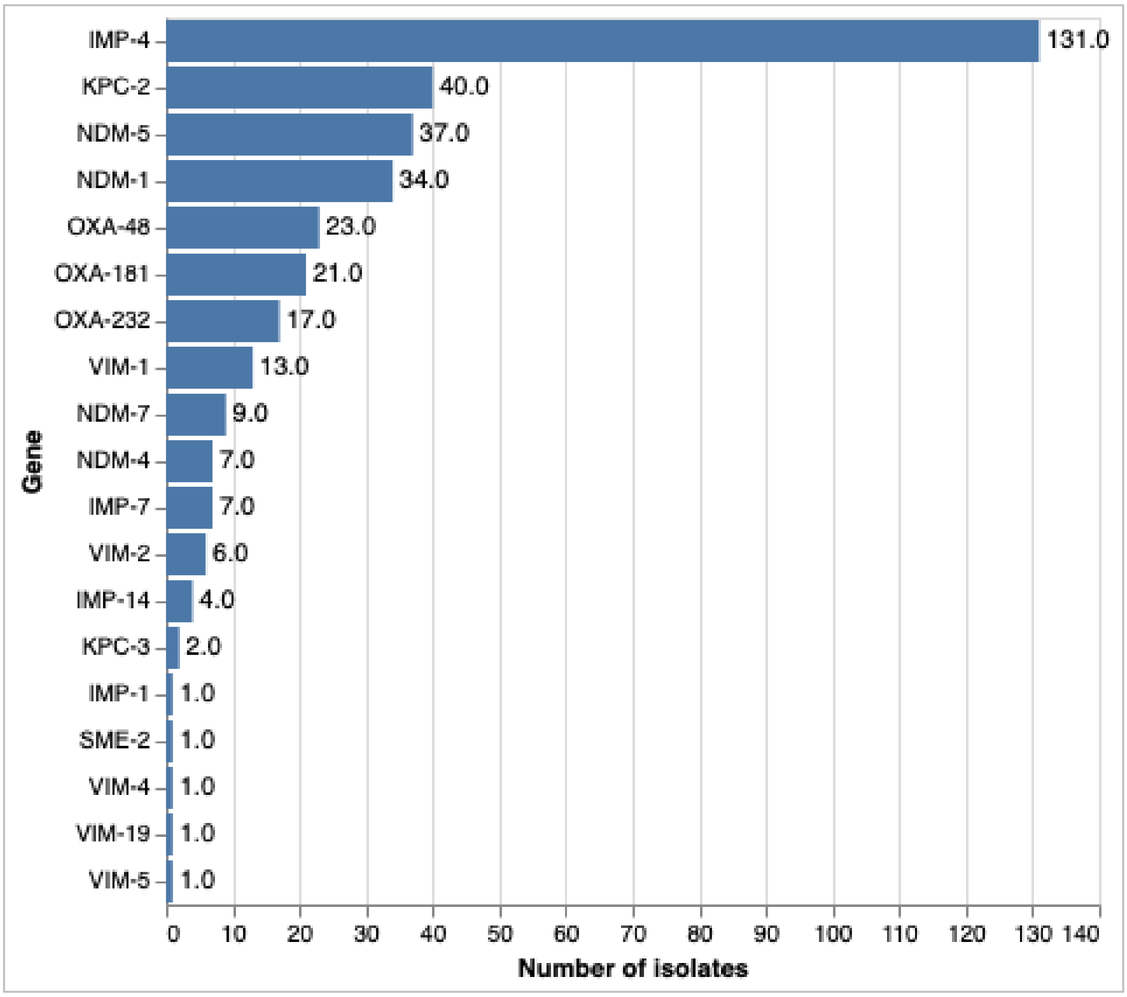

Source data are provided as a Source Data file.

Supplementary Figure 8. Genera and species included in validation of resistance allele calling

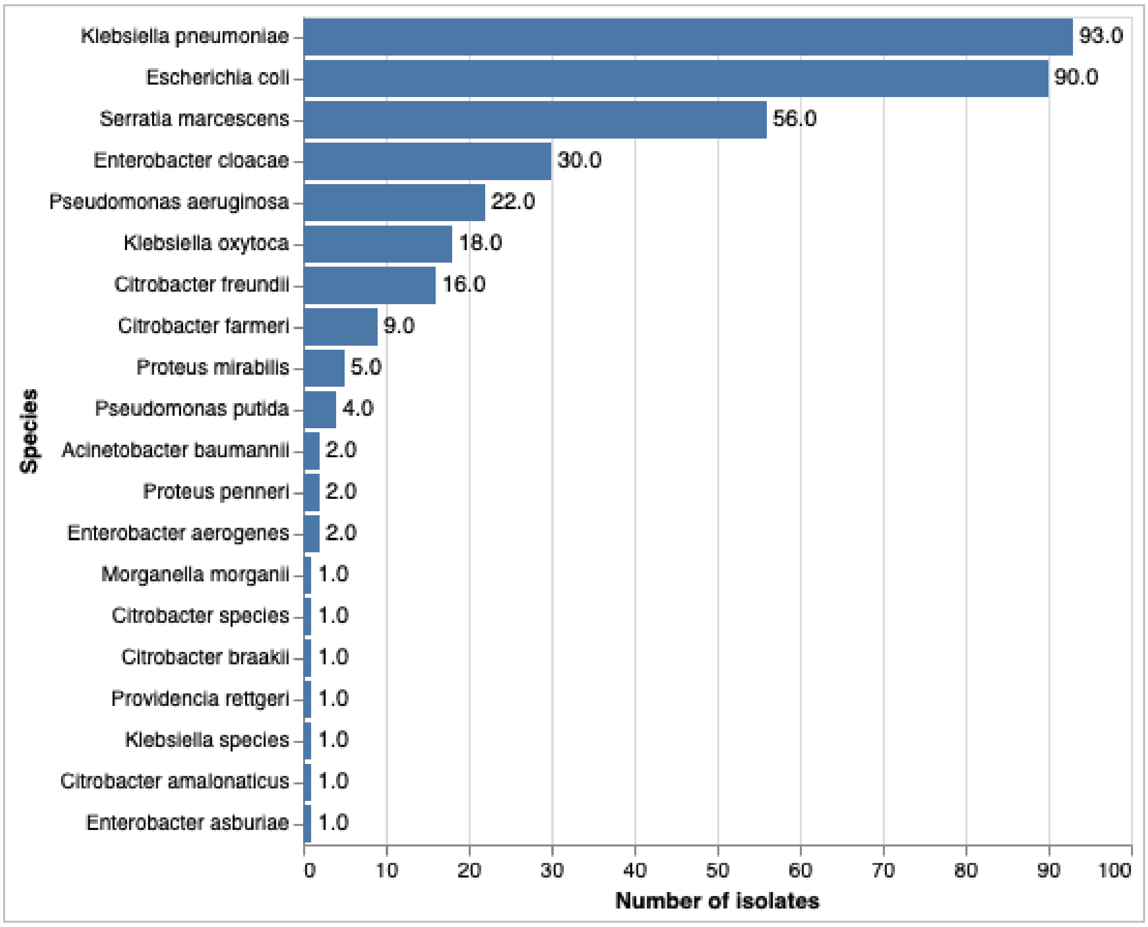

Source data are provided as a Source Data file.

Supplementary Figure 9. Genera and species included in validation of resistance allele calling (synthetic dataset)

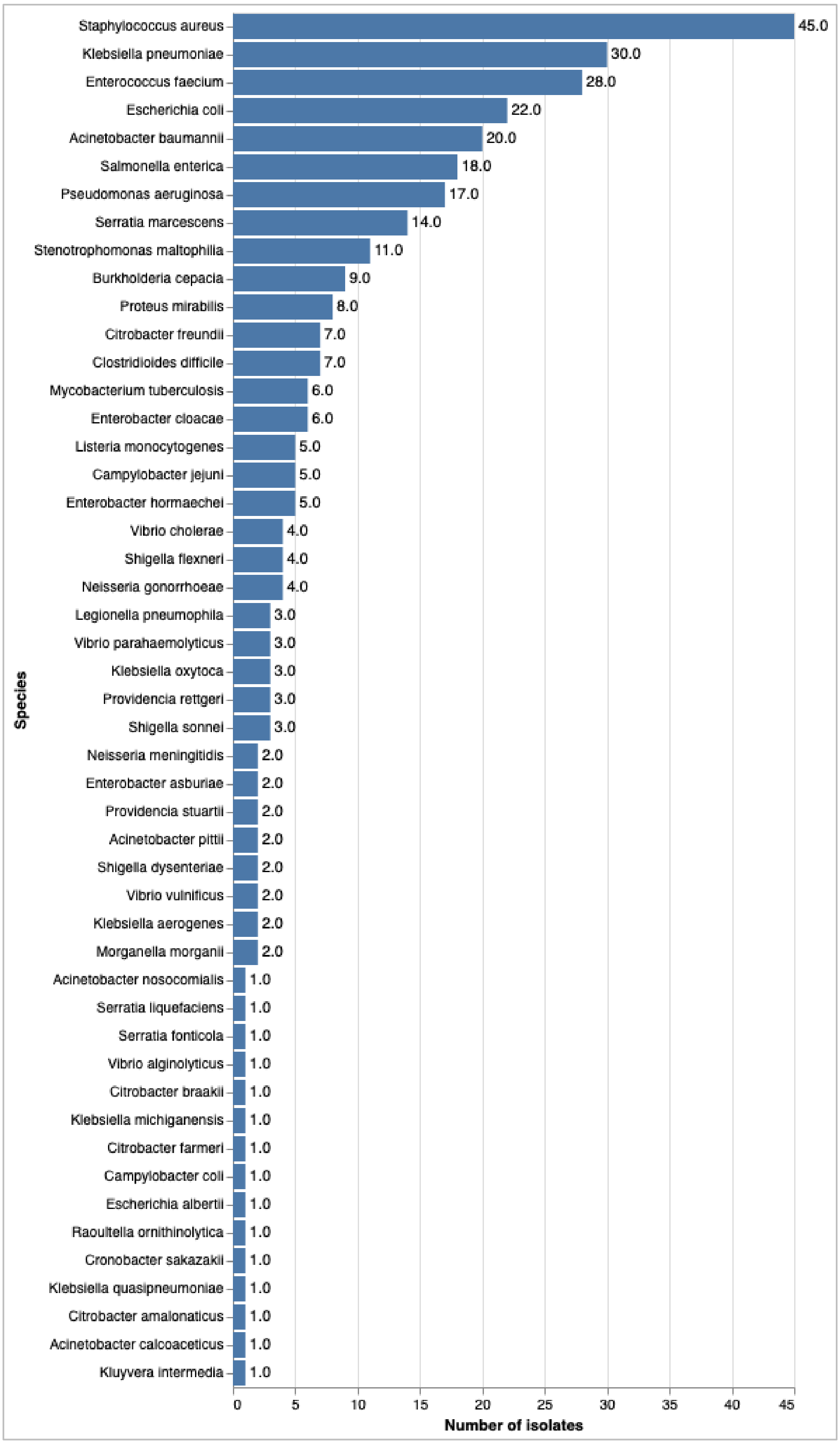

Source data are provided as a Source Data file.

Supplementary Figure 10. Drug classes with represented in synthetic dataset for the validation of resistance allele calling

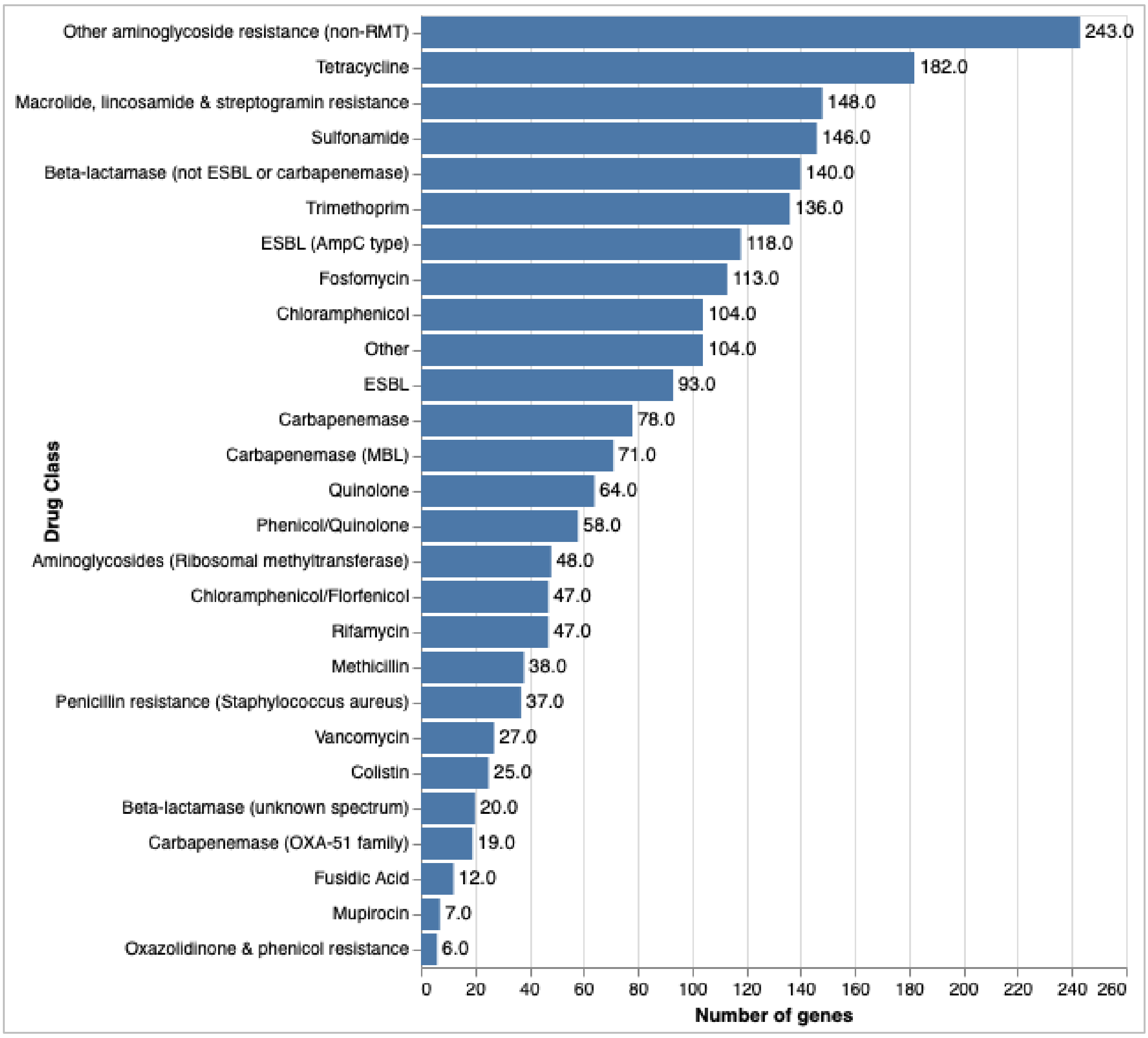

Source data are provided as a Source Data file.

Supplementary Table 1. Performance of *abritAMR* on synthetic dataset using different genome assembly tools

Genome assembly tool	Accuracy (%)	Sensitivity (%)	Specificity (%)	PPV (%)	NPV (%)
Shovill [1]	99.93	97.15	100	99.98	99.93
SPAdes [2]	99.93	97.22	100	99.98	99.93
SKESA [3]	99.88	95.23	100	100	99.88

PPV, positive predictive value; NPV, negative predictive value.

Supplementary Table 2. Validation of inferred phenotype for *Salmonella* spp. by *abritAMR*[a]

Antimicrobial	Accuracy[b] (%, 95%CI)	Sensitivity (%, 95%CI)	Specificity (%, 95%CI)	PPV (%, 95%CI)	NPV (%, 95%CI)
**Ampicillin (*n*=863)**	99.1 (98.2-99.6)	99.2 (97.8-99.8)	98.9 (97.5-99.7)	98.7 (97.1-99.6)	99.4 (98.1-99.9)
**Cefotaxime (*n*=862)**	99.9 (99.4-100)	100 (92.0-100)	99.9 (99.3-100)	97.8 (88.2-99.9)	100 (99.5-100)
**Meropenem[c] (*n*=789)**	100 (99.5-100)	100 (15.8-100)	100 (99.5-100)	100 (15.8-100)	100 (99.5-100)
**Gentamicin[d] (*n*=846)**	99.8 (99.1-100)	100 (87.2-100)	99.8 (99.1-100)	93.1 (77.2-99.2)	100 (99.5-100)
**Kanamycin[d] (*n*=864)**	100 (99.6-100)	100 (92.3-100)	100 (99.6-100)	100 (92.3-100)	100 (99.6-100)
**Streptomycin[d] (n=716)**	95.5 (93.7-96.9)	99.2 (97.0-99.9)	93.7 (91.1-95.7)	88.8 (84.5-92.3)	99.6 (98.4-99.9)
**Sulfathiazole (*n*=864)**	98.8 (97.9-99.4)	97.9 (95.7-99.2)	99.4 (98.4-99.9)	99.1 (97.4-99.8)	98.7 (97.3-99.5)
**Trimethoprim (*n*=864)**	99.5 (98.8-99.9)	100 (97.5-100)	99.4 (98.6-99.8)	97.4 (93.4-99.3)	100 (99.5-100)
**Trimethoprim-Sulfamethoxazole (*n*=864)**	99.1 (98.2-99.6)	96.9 (92.3-99.1)	99.5 (98.6-99.9)	96.9 (92.3-99.1)	99.5 (98.6-99.9)
**Tetracycline (*n*=859)**	98.5 (97.4-99.2)	99.4 (98.0-99.9)	97.8 (96.1-98.9)	97.0 (94.7-98.5)	99.6 (98.5-100)
**Chloramphenicol (*n*=858)**	99.3 (98.5-99.7)	100 (96.7-100)	99.2 (98.3-99.7)	94.8 (89.0-98.1)	100 (99.5-100)
**Azithromycin (*n*=864)**	99.2 (98.3-99.7)	85.3 (68.9-95.0)	99.8 (99.1-100)	93.5 (78.6-99.2)	99.4 (98.6-99.8)
**Ciprofloxacin[e] (*n*=864)**	96.8 (95.4-97.8)	99.6 (98.5-99.9)	93.5 (90.6-95.7)	94.7 (92.3-96.5)	99.5 (98.1-99.9)
**Summary performance (all antimicrobials)(95% CI)**	**98.9 (98.7-99.1)**	**98.9 (98.4-99.3)**	**98.9 (98.7-99.1)**	**96.1 (95.2-96.8)**	**99.7 (99.6-99.8)**

PPV, positive predictive value; NPV, negative predictive value; CI, confidence interval.

[a] Compared to agar dilution using CLSI methods and breakpoints (CLSI M100 2020). Excludes all 'intermediate' results from AST.

[b] Accuracy determined by number of true positives and negatives divided by total number of results (true positive, genotypic and phenotypic resistance; true negative, no AMR mechanisms detected in phenotypically susceptible isolate).

[c] Note only two carbapenemase-producing isolates were available for inclusion in the validation dataset.

[d] Note that aminoglycosides are not used for patient treatment, but included here for surveillance purposes.

[e] For ciprofloxacin, 'true positive' defined as concordant intermediate or resistant results (phenotype and genotype).

Supplementary Table 3. Resistance classes and gene targets determined using synthetic reads

AMRFinderPlus Class	*AMRFinderPlus* Subclass	*abritAMR* Enhanced Subclass	Grouping for reporting	Included in validation panel?
Aminoglycoside	By gene: *armA, npmA,* and all *rmt* alleles	Aminoglycoside resistance (ribosomal methyltransferases)	Aminoglycoside resistance (ribosomal methyltransferases)	Y
	Amikacin/Gentamicin/ Kanamycin/Tobramycin	Amikacin/Gentamicin/ Kanamycin/Tobramycin	Other aminoglycoside resistance	Y
	Amikacin/Kanamycin	Amikacin/Kanamycin		Y
	Amikacin/Kanamycin/Tobramycin	Amikacin/Kanamycin/Tobramycin		Y
	Amikacin/Quinolone	Amikacin/Quinolone		Y
	Amikacin/Tobramycin	Amikacin/Tobramycin		N
	Aminoglycoside	Aminoglycoside		Y
	Gentamicin	Gentamicin		Y
	Gentamicin/Kanamycin/Tobramycin	Gentamicin/Kanamycin/Tobramycin		Y
	Gentamicin/Tobramycin	Gentamicin/Tobramycin		Y
	Hygromycin	Hygromycin		Y
	Kanamycin	Kanamycin		Y
	Kanamycin/Tobramycin	Kanamycin/Tobramycin		Y
	Kasugamycin	Kasugamycin		N
	Spectinomycin	Spectinomycin		Y
	Streptomycin	Streptomycin		Y
	Streptomycin/Spectinomycin	Streptomycin/Spectinomycin		Y
	Tobramycin	Tobramycin		Y
Beta-lactam	Carbapenem	Carbapenemase (MBL)<br>Carbapenemase (OXA-51 family)<br>Carbapenemase (see Appendix 15.1 for classification rules)	Carbapenemase (MBL)<br>Carbapenemase (OXA-51 family)<br>Carbapenemase	Y

AMRFinderPlus Class	*AMRFinderPlus* Subclass	*abritAMR* Enhanced Subclass	Grouping for reporting	Included in validation panel?
	Cephalosporin	ESBL<br>ESBL (AmpC)<br>Beta-lactamase (not carbapenemase or ESBL)<br>(see Appendix 15.1 for classification rules)	ESBL<br>ESBL (AmpC)<br>Beta-lactamase (not carbapenemase or ESBL)	Y
	Beta-lactam	Beta-lactamase (not carbapenemase or ESBL)<br>Beta-lactamase (unknown spectrum)<br>Penicillin resistance (*Staphylococcus aureus*) (see Appendix 15.1 for classification rules)	Beta-lactamase (not carbapenemase or ESBL)<br>Beta-lactamase (unknown spectrum)<br>Penicillin resistance (*Staphylococcus aureus*)	Y
	Cephalothin	ESBL (AmpC type)	ESBL (AmpC type)	Y
	Methicillin	Methicillin	Methicillin	Y
Colistin	Colistin	Colistin	Colistin	Y
Fosfomycin	Fosfomycin	Fosfomycin	Fosfomycin	Y
Fusidic Acid	Fusidic Acid	Fusidic Acid	Fusidic Acid	Y
Glycopeptide	Vancomycin	Vancomycin	Vancomycin	Y
Lincosamide	Lincosamide	Lincosamide	Macrolide, lincosamide and/or streptogramin resistance	Y
Lincosamide/Streptogramin	Lincosamide/Streptogramin	Lincosamide/Streptogramin	Macrolide, lincosamide and/or streptogramin resistance	Y
Macrolide	Macrolide	Macrolide	Macrolide, lincosamide and/or streptogramin resistance	Y
	Erythromycin	Erythromycin	Macrolide, lincosamide and/or streptogramin resistance	N
	Erythromycin/Telithromycin/Tylosin	Erythromycin/Telithromycin/Tylosin	Macrolide, lincosamide and/or streptogramin resistance	N
Macrolide/Lincosamide/Streptogramin	Macrolide/Lincosamide/Streptogramin	Macrolide/Lincosamide/Streptogramin	Macrolide, lincosamide and/or streptogramin resistance	Y
Macrolide/Pleuromutilin	Lincosamide/Streptogramin/Tiamulin	By gene: *cfr* alleles, *cipA, clbA*: Oxazolidinone & phenicol resistance	Oxazolidinone & phenicol resistance	N

AMRFinderPlus Class	*AMRFinderPlus* Subclass	*abritAMR* Enhanced Subclass	Grouping for reporting	Included in validation panel?
Streptogramin	Streptogramin	Streptogramin	Macrolide, lincosamide and/or streptogramin resistance	N
Mupirocin	Mupirocin	Mupirocin	Mupirocin	Y
Nitroimidazole	Nitroimidazole	Nitroimidazole	Nitroimidazole	N
Phenicol	Chloramphenicol	Chloramphenicol	Chloramphenicol	Y
	Chloramphenicol/Florphenicol	Chloramphenicol/Florphenicol	Chloramphenicol/Florphenicol	Y
	Phenicol	Phenicol	Phenicol	N
Phenicol/ Oxazolidinone	Florfenicol/Oxazolidinone	Oxazolidinone & phenicol resistance (by gene: *optrA*)	Oxazolidinone & phenicol resistance	Y
Phenicol/Quinolone	Phenicol/Quinolone	Phenicol/Quinolone	Phenicol/Quinolone	Y
Quinolone	Quinolone	Quinolone	Quinolone	Y
Rifamycin	Rifampin	Rifampin	Rifampin	Y
Sulfonamide	Sulfonamide	Sulfonamide	Sulfonamide	Y
Tetracycline	Tetracycline	Tetracycline	Tetracycline	Y
Trimethoprim	Trimethoprim	Trimethoprim	Trimethoprim	Y
Avilamycin	Avilamycin	Other	Other	Y
Bleomycin	Bleomycin, Zorbamycin	Other	Other	Y
Pleuromutilin	Tiamulin	Other	Other	N
Streptothricin	Streptothricin	Other	Other	Y
Tetracenomycin	Tetracenomycin	Other	Other	Y
Tuberoactinomycin	Viomycin	Other	Other	N
Thiostrepton	Thiostrepton	Other	Other	N

Note: AMR gene groups not included in the validation panel were unable to be readily identified in RefSeq genomes of the priority species for our laboratory to validate (aiming to primarily include species commonly sequenced in our public health laboratory work)

Supplementary Table 4. Steps and logic for *abritAMR* Classification Database updates

After each new *AMRFinderPlus* release (those including new AMR genes or mutations), the *abritAMR* Classification Database is updated by the curators.

An initial classification step is performed according to the following logic for all new AMR genes added in the database update, as described in Table S2 below. This is supplemented by manual review and curation of critical resistance gene families, and other groups not covered by the update logic. Additionally, any changes in existing genes since the last update are reviewed.

**Step 1: Classification by alleles**	
***AMRFinderPlus* allele**	***abritAMR* Classification Database Enhanced Subclass**
rmt, armA, npmA*	Aminoglycoside resistance (ribosomal methyltransferases)
optrA, cfr, cipA, clb* alleles	Oxazolidinone & phenicol resistance
**Step 2: Classification by NCBI Subclass**	
***AMRFinderPlus* Subclass**	***abritAMR* Classification Database Enhanced Subclass**
Carbapenemase	If "metallo" in description → Carbapenemase (MBL);<br>If "OXA-51 family" or allele=OXA-51 → "Carbapenemase (OXA-51 family);<br>Otherwise "Carbapenemase"
Cephalosporin	If description includes "class C" → "ESBL (AmpC)";<br>If description includes "extended-spectrum" and NOT "class C" → "ESBL"
Beta-lactam	If description includes "broad-spectrum" or "carbenicillin-hydrolyzing" → classified as "Beta-lactamase (not ESBL or carbapenemase)";<br>If *blaZ** alleles -> "Penicillin resistance (*Staphylococcus aureus*)"<br>Other groups curated curated manually; if no phenotype found, called "Beta-lactamase (unknown spectrum)"
Colistin	Colistin
Vancomycin	Vancomycin
Methicillin	Methicillin
Fosfomycin	Fosfomycin
Quinolone	Quinolone
Tetracycline	Tetracycline
Trimethoprim	Trimethoprim
Rifamycin	Rifamycin
Chloramphenicol	Chloramphenicol
Chloramphenicol/Florfenicol	Chloramphenicol/Florfenicol
Phenicol	Phenicol
Phenicol/Quinolone	Phenicol/Quinolone
Fusidic acid	Fusidic acid

Mupirocin	Mupirocin
Nitroimidazole	Nitroimidazole
Amikacin/Gentamicin/Kanamycin/Tobramycin	Other aminoglycoside resistance (non-RMT)
Amikacin/Kanamycin	Other aminoglycoside resistance (non-RMT)
Amikacin/Kanamycin/Tobramycin	Other aminoglycoside resistance (non-RMT)
Amikacin/Quinolone	Other aminoglycoside resistance (non-RMT)
Amikacin/Tobramycin	Other aminoglycoside resistance (non-RMT)
Aminoglycosides	Other aminoglycoside resistance (non-RMT)
Gentamicin	Other aminoglycoside resistance (non-RMT)
Gentamicin/Kanamycin/Tobramycin	Other aminoglycoside resistance (non-RMT)
Gentamicin/Tobramcyin	Other aminoglycoside resistance (non-RMT)
Kanamycin	Other aminoglycoside resistance (non-RMT)
Kanamycin/Tobramycin	Other aminoglycoside resistance (non-RMT)
Spectinomycin	Other aminoglycoside resistance (non-RMT)
Streptomycin	Other aminoglycoside resistance (non-RMT)
Streptomycin/Spectinomycin	Other aminoglycoside resistance (non-RMT)
Tobramycin	Other aminoglycoside resistance (non-RMT)
Erythromycin	Macrolide, lincosamide & streptogramin resistance
Erythromycin/Telithromycin	Macrolide, lincosamide & streptogramin resistance
Lincosamides	Macrolide, lincosamide & streptogramin resistance
Lincosamides/Streptogramin	Macrolide, lincosamide & streptogramin resistance
Macrolides	Macrolide, lincosamide & streptogramin resistance
Streptogramin	Macrolide, lincosamide & streptogramin resistance
Bleomycin	Other
Pleuromutilin	Other
Avilamycin	Other
Streptothricin	Other
Tetracenomycin	Other
Thiostrepton	Other
Tuberactinomycin	Other

Step 3: Manual review and curation

***AMRFinderPlus* Subclass**	***abritAMR* Classification Database Enhanced Subclass**
Carbapenemase, Cephalosporin, Beta-lactam	Classifications reviewed and curated manually<br>Where phenotype unclear from gene family or description, manual search to determine appropriate subclass:<br>• Review GenBank reference sequence record for associated publications and references<br>• Search CARD for record or publication<br>• Search PubMed and Google Scholar for publications<br><br>When no phenotypic data available (insufficient description, no publications, or metagenomic data), classify as 'Beta-lactamase (unknown spectrum)'

**Step 4: Review changes to existing genes since last update**
Any genes where the class, subclass or description have changed since the last update are also reviewed to determine the significance, and if any changes are required to the Enhanced Subclass

*, wildcard (i.e. any character/s); RMT, ribosomal methyltransferase; CARD, Comprehensive Antimicrobial Resistance Database (https://card.mcmaster.ca).

Supplementary Table 5. Gene targets and PCR primers for carbapenemase and ESBL detection and allelic typing by Sanger sequencing

Gene/Target	Primer	Primer sequence	Product size (bp)	Ref
blaKPC	KpcF<br>KpcR	ATG TCA CTG TAT CGC CGT C<br>TTA CTG CCC GTT GAC GCC-3'	845	4
blaOXA-48-like	OXA-48F<br>OXA-48R	TTG GTG GCA TCG ATT ATC GG<br>GAG CAC TTC TTT TGT GAT GGC	743	5
blaIMP	IMP-A<br>IMP-B	GAA GGY GTT TAT GTT CAT AC<br>GTA MGT TTC AAG AGT GAT GC	587	6
blaVIM	VIM2004-A<br>VIM2004-B	GTT TGG TCG CAT ATC GCA AC<br>AAT GCG CAG CAC CAG GAT AG	382	
blaNDM	NDM-F<br>NDM-R	GGG CAG TCG CTT CCA ACG GT<br>GTA GTG CTC AGT GTC GGC AT	475	7
IMP sequencing 5'CS-IMP-B	5'CS<br>IMP-B	GGC ATC CAA GCA GCA AG<br>GTA MGT TTC AAG AGT GAT GC	793	6
VIM 1 amplification & sequencing	Vim-1F<br>Vim-1R	TTA TGG AGC AGC AAC GAT GT<br>CAA AAG TCC CGC TCC AAC GA	920	8
VIM-2 amplification & sequencing	Vim-2F<br>Vim-2R	AAA GTT ATG CCG CAC TCA CC<br>TGC AAC TTC ATG TTA TGC CG	865	
VIM-2 seq	Vim-2sF<br>Vim-2sR	TCG ACG GTG ATG CGT ACG TT<br>TTG ATG TCC TTC GGG CGG CT	865	
NDM seq	NDMLF<br>NDMLR	ATG GAA TTG CCC AAT ATT ATG CAC<br>TCA GCG CAG CTT GTC GGC	813	9
blaMOX	MOXF<br>MOXR	GCT GCT CAA GGA GCA CAG GAT<br>CAC ATT GAC ATA GGT GTG GTG C	520	10
blaCIT	CITF<br>CITR	TGG CCA GAA CTG ACA GGC AAA<br>TTT CTC CTG AAC GTG GCT GGC	462	
blaDHA	DHAF<br>DHAR	AAC TTT CAC AGG TGT GCT GGG T<br>CCG TAC GCA TAC TGG CTT TGC	405	
blaACC	ACCF<br>ACCR	AAC AGC CTC AGC AGC CGG TTA<br>TTC GCC GCA ATC ATC CCT AGC	346	
blaEBC	EBCF<br>EBCR	TCG GTA AAG CCG ATG TTG CGG<br>CTT CCA CTG CGG CTG CCA GTT	302	
blaFOX	FOXF<br>FOXR	AAC ATG GGG TAT CAG GGA GAT G<br>CAA AGC GCG TAA CCG GAT TGG'	190	
blaOXA-23-like	OXA-23F<br>OXA-23R	GAT CGG ATT GGA GAA CCA GA<br>ATT TCT GAC CGC ATT TCC AT	5001	11
blaOXA-24-like	OXA-24F<br>OXA-24R	GGT TAG TTG GCC CCC TTA AA<br>AGT TGA GCG AAA AGG GGA TT	246	
blaOXA-51-like	OXA-51F<br>OXA-51R	TAA TGC TTT GATCGG CCT TG<br>TGG ATT GCA CTT CAT CTT GG	353	
blaOXA-58-like	OXA-58F<br>OXA-58R	AAG TAT TGG GGC TTG TGC TG<br>CCC CTC TGC GCT CTA CAT AC	599	

Supplementary Table 6. Isolates and sequence runs used to determine precision

Species	Average genome length Mbp (Range)	Average % GC (Range)	Gram stain
Clostridium difficile	4.23 (4.05-4.46)	28.81 (28.35-29.20)	Positive
Campylobacter jejuni	1.67 (1.61-1.85)	30.41 (30.18-30.70)	Negative
Listeria monocytogenes	2.96 (2.78-3.24)	38.02 (37.86-38.30)	Positive
Legionella pneumophila	3.43 (2.68-3.79)	38.34 (38.10-38.60)	Negative
Streptococcus pyogenes	1.84 (1.70-1.95)	38.51 (38.20-38.70)	Positive
Escherichia coli	5.07 (4.48-5.87)	50.69 (50.29-51.20)	Negative
Neisseria meningitidis	2.19 (2.14-2.32)	51.65 (51.30-51.90)	Negative
Salmonella Typhimurium	4.82 (4.76-5.18)	52.15 (52.08-52.30)	Negative
Neisseria gonorrhoeae	2.21 (2.15-2.24)	52.48 (52.40-52.70)	Negative
Klebsiella pneumoniae	5.6 (5.09-6.13)	57.16 (56.54-58.00)	Negative
Mycobacterium tuberculosis	4.41 (4.38-4.44)	65.60 (65.60-65.60)	(Positive)
Pseudomonas aeruginosa	6.62 (6.12-7.50)	66.24 (65.60-66.70)	Negative
Burkholderia lata	7.62 (6.42-8.61)	66.84 (66.60-67.06)	Negative

Supplementary Methods

Summary of accredited genomic sequencing workflow, Microbiological Diagnostic Unit Public Health Laboratory (MDU PHL)

This process covers whole genome sequencing (WGS) from isolate to generation of FASTQ files for bioinformatic analysis. This workflow is designed for high-throughput genomic sequencing using robotic liquid handling equipment.

Overview of accredited genomic sequencing workflow

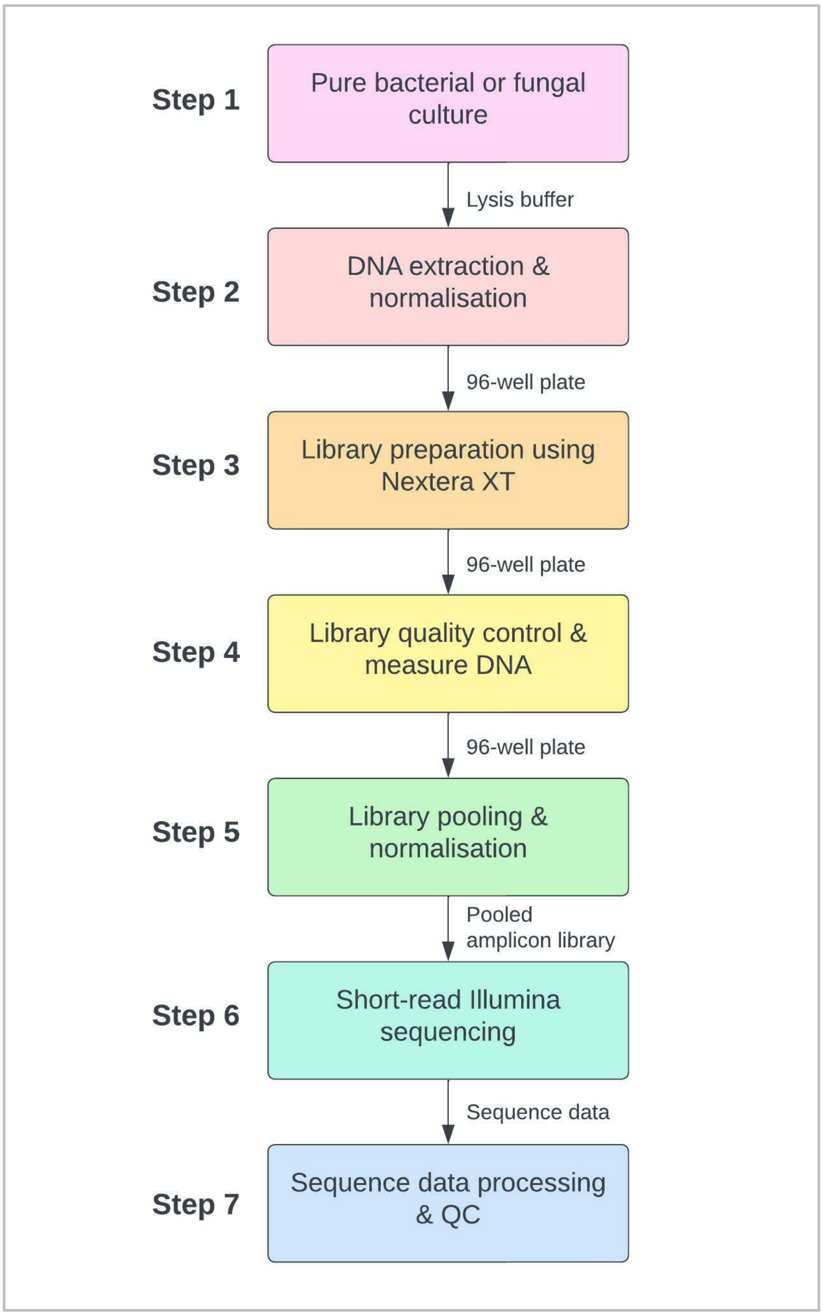

Test accreditation: ISO15189 Medical, ISO17025 Biological & Veterinary

STEP 1

Workflow inputs: Pure isolates of bacteria, or extracted total nucleic acid (TNA) referred by external organisations (if extracted TNA, maintain at 4°C on arrival, or freeze -20°C if not processed within 24h).

Controls:

- *Enterococcus faecium* (AUS0085; NCBI:txid1305849, Lam et al. 2013 [12]) and uninoculated lysis buffer; both controls used from extraction through to sequencing
- PhiX v3 (FC-110-3001; Illumina)– commercially prepared sequencing library specifically for sequencing component added at a final concentration of 1.8pM.

Pick-off from purity plate:

1. Briefly, colony picks are prepared by taking a 1µl loopful of bacteria from a 24hr culture and emulsifying into 100µl of Gram-positive lysis buffer (20mM Tris-HCL, pH 8.0, 2mM EDTA, 1.2% TritonX-100) in a 2ml microfuge tube. Where possible a single bacterial colony is selected, or 2-5 colonies if colonies are small.
2. Add 100 µl Gram positive lysis buffer (20mM Tris-HCL, pH 8.0, 2mM EDTA, 1.2% TritonX-100, 40 mg/ml lysozyme). For *Salmonella*, use 200 µl lysis buffer and omit step 2.
3. Incubate 30 minutes at 37°C.
4. Continue with DNA extraction procedure (at MDU PHL, on the QIAsymphony using the QIAsymphony DSP Virus/Pathogen Mini kit and protocol Complex200_V6_DSP protocol). Alternatively, manual extractions can be performed using the QIAamp DNA Mini kit using buffer EB for elution.

STEP 2

DNA extraction & normalisation:

- Maximum 96 samples in a single DNA extraction run (94 test samples and 2 controls).
- DNA extraction (using paramagnetic bead DNA/RNA extraction, on QIAsymphony (Qiagen) or Chemagic 360 (Perkin Elmer) at MDU PHL) or alternative validated protocol. DNA must be eluted in either sterile RNase/DNase free water or a low salt, EDTA free elution buffer provided by the manufacturer of the DNA extraction kit (no EDTA).
- Measure concentration of gDNA using the Quant-iT assay (Qubit assay) or alternative method. Minimum concentration of 0.15ng/µl is required to proceed to library preparation (note: the NexteraXT kit is optimized for 1ng of input DNA). Ideal DNA concentration after DNA extraction is 1-15ng/µl.

STEP 3

Library preparation (Nextera XT):

- Prepare sequencing libraries using 5µl of normalized gDNA with the NexteraXT library preparation kits (FC-131-1096) https://sapac.support.illumina.com/sequencing/sequencing_kits/nextera_xt_dna_kit/documentation.html – this includes tagmentation of input DNA, PCR amplification and PCR

clean-up according to manufacturer's instructions (can be manual or automated by robotic liquid handlers).

- Assign an index set (FC-131-2001, FC-131-2002, FC131-2003, FC-131-2004) for each library. At MDU PHL, data is managed using the commercial LIMS system ClarityLIMS (Illumina) to capture batch number and operator information in addition to quality metrics.

STEP 4

Library quality control & measurement of DNA concentration:

- Perform QC of the library on the LabchipGX Touch using a DNA 5K/RNA/CZE LabChip (Cat no. 760435) using the Quant-iT kit or TapeStation using a D5000 Screen tape (5067-5588) using the D5000 reagents (Cat no. 5067-5589)and. Record the library fragment size and concentration data.
- Calculate the molarity of each library using the Molarity Calculation step in ClarityLIMS to convert ng/µl to nM according to the following formula:

(ng/µl)/ (660 g/mol x average library size in bp) x 106 = concentration in nM

- Create a Basespace sample sheet using data extracted from the 'PCR amplification' step in ClarityLIMS.

STEPS 5 & 6

Library pooling, normalisation and sequencing:

- Libraries are pooled for sequencing on an instrument (NextSeq500 or NextSeq550). Choice of sequencing cartridges is dependent on the number of samples to be sequenced.

- For NextSeq 500/550:

**Sequencing cartridge**	**Sample no. (based on *Salmonella* genome size and >70X coverage)**
2 x 150bp mid-output (300 cycles)	94
2 x 150bp high-output (300 cycles)	275

- Perform library normalization to 4nM to produce the pooled amplicon library (PAL). This uses the expected species (to calculate relative genome size), clean amplified nucleic acid (CAN) concentration, size of library and molarity of CAN to calculate the volume of each library and diluent required to make up the PAL.
- Always include the *E. faecium* (PTC), uninoculated lysis buffer (NTC) and PhiX is spiked into the sequencing pool
- Quality control – repeat library preparation if insert size <200bp. or if NTC generates a library >1M reads and contamination is assessed to impact all samples on the run. PTC data may be used to monitor reagents and sample processing steps and PhiX is used to assess instrument performance and troubleshooting, and to generate trend data on accuracy and reproducibility of sequences.

Library preparation troubleshooting:

Problem	Solution
Inconsistent DNA quantitation using Quant-iT assay	Ensure standards are in date. Ensure the sample and working solution are adequately mixed and at room temperature. Check elution buffer is EDTA free and low salt
DNA insert sizes are <200bp	This may happen if tagmentation step is >10 mins or DNA concentration is too low, repeat if necessary
Library does not reach 4nM	Start with a higher concentration of DNA. Check the quality of the DNA. Check the elution buffer (inhibitors for library prep)

- Sequence the pooled amplicon library as per manufacturer's instructions for the selected instrument. Ensure the PhiX control library is included.
- Prepare a sample sheet for de-multiplexing BCL files

STEP 7

Quality of control of sequencing results

A. Sequence run QC: parameters depend on the sequencer and cartridge used. Examples below for NextSeq 500/550:

- >Q30 (usually >75%)
- Cluster density (usually 170-220K/mm^2)
- Clusters passing filter (NextSeq500/550: >80% (<260M) for mid-output, >85% (<800M) for high-output kit)
- Estimated yield (32-39Gb for mid-output, 100-120Gb for high-output)

PhiX control: Used for QC of the sequencing process and to add diversity into the sequencing pool when sequencing a number of clonal or highly-related organisms. If all libraries fail to sequence but PhiX sequences adequately, this suggests library failure, whereas failure of all libraries plus PhiX suggests complete run failure at the sequencing stage. A PhiX control run can also be used independently to check the performance of the sequencer. For the NextSeq 500/550, 100% PhiX should be loaded at 1.8pM and optimal raw cluster densities should range from 170-220K clusters/mm^2.

PhiX loading concentrations for validation runs on Illumina platforms

Platform	Optimal Loading Concentration	Optimal Raw Cluster Density
iSeq100	100 pM	N/A*
MiniSeq	1.4 pM	170-220K clusters/mm^2
MiSeq v2 reagents	12.5 pM	1000-1200K clusters/mm^2
MiSeq v3 reagents	20 pM	1200-1400K clusters/mm^2
NextSeq™ 500/550 High Output reagents	1.5 pM	170-220K clusters/mm^2
NextSeq 500/550 Mid Output reagents	1.5 pM	170-220K clusters/mm^2
NextSeq 1000/2000	650 pM	N/A*

*Patterned flow cells consist of a nanowell with ordered wells, no variation in reported cluster density from run to run

Refer to the web site: https://support.illumina.com/bulletins/2016/10/phix-loading-concentrations-for-validation-runs-on-illumina-sequencing-platforms.html

B. Quality control of reads (bioinformatics)

QC metrics include assessment of sequencing metrics for each run and each sequence

Metrics applied to each sequence:

- Yield: average estimated read depth (coverage) ≥40X (estimated using KMC)
- Sequence quality: average Q-score≥30 (using seqtk fqchk)
- Compare species detected from reads (*k*-mer identification using kraken2) with the species expected (based on information supplied at time of sequencing). These sequences are flagged for manual review by the submitting wet lab team. Inconsistencies may be due to:
    - *Laboratory error* – if sequence providence cannot be confirmed – fails QC. Subsequent investigations of sample by lab may lead to manual pass (e.g. MALDI-ToF identification consistent with WGS)
    - *Contamination or mixed sequence* – fail QC
    - Additionally, consistency between dominant species assembled (based on appropriate ST scheme) is considered in order to confirm that species is as expected.
- Genome size – genome size based on *k*-mers (KMC); assembled genome size should be within expected range of the species detected (±10%) based on a collection of public genomes (https://www.ncbi.nlm.nih.gov/genome/browse/reference/)
- MLST – sequence type detected (where a scheme exists for the detected species)

Additional metrics are examined to assess the performance of the sequencing run as a whole:

- Assess the proportion of 'Undetermined' reads per sequencing run – a higher than 5% proportion may indicate an issue with input data in the form of sample sheet errors
- Performance of controls
    - Negative control - fail if >1x10^6 reads are present; re-examine plate for evidence of cross-well contamination; consider re-sequencing whole plate if possible contamination
    - Positive control (*E. faecium*) – expect ST203, (scheme: *efaecium*), genome size (2.57-3.40MB), minimum yield $40*2.57*10^6$. Fail if doesn't meet individual sequence metrics above; QC pipeline stops and prompt bioinformatician to examine other features of the sequencing run
    - PhiX control –used for troubleshooting in the event of complete run failure
- If more than 25% of a sequencing run fails due to a mismatch between species expected and species observed, QC pipeline stops and further investigation by laboratory staff is undertaken before bioinformatics proceeds with the run. This may be indicative of an issue in the lab (plates swapped around, library prep issues).
- Quality assessment of genome assemblies (e.g. number of contigs or N50) is not usually required as sequencing validation and accreditation was developed to ensure optimal assemblies for the purpose of gene detection. Specifically, the genome size of an assembled genome must be within the expected range with a valid ST detected (where a scheme exists).

Supplementary References

1. Seemann T. Shovill: assemble bacterial isolate genomes from Illumina paired-end reads. GitHub; 2017.
2. Bankevich A, Nurk S, Antipov D, Gurevich AA, Dvorkin M, Kulikov AS, Lesin VM, Nikolenko SI, Pham S, Prjibelski AD. SPAdes: a new genome assembly algorithm and its applications to single-cell sequencing. *J Comput Biol* 2012; **19**(5): 455-77.
3. Souvorov A, Agarwala R, Lipman DJ. SKESA: strategic k-mer extension for scrupulous assemblies. *Genome Biol* 2018; **19**(1).
4. Endimiani A, Carias LL, Hujer AM, Bethel CR, Hujer KM, Perez F, Hutton RA, Fox WR, Hall GS, Jacobs MR. Presence of plasmid-mediated quinolone resistance in *Klebsiella pneumoniae* isolates possessing bla_{KPC} in the United States. *Antimicrob Agents Chemother* 2008; **52**(7): 2680-2.
5. Poirel L, Héritier C, Tolün V, Nordmann P. Emergence of oxacillinase-mediated resistance to imipenem in *Klebsiella pneumoniae*. *Antimicrob Agents Chemother* 2004; **48**(1): 15-22.
6. Pitout JD, Gregson DB, Poirel L, McClure J-A, Le P, Church DL. Detection of *Pseudomonas aeruginosa* producing metallo-β-lactamases in a large centralized laboratory. *J Clin Microbiol* 2005; **43**(7): 3129-35.
7. Mushtaq S, Irfan S, Sarma J, Doumith M, Pike R, Pitout J, Livermore D, Woodford N. Phylogenetic diversity of *Escherichia coli* strains producing NDM-type carbapenemases. *J Antimicrob Chemother* 2011; **66**(9): 2002-5.
8. Yan J-J, Hsueh P-R, Ko W-C, Luh K-T, Tsai S-H, Wu H-M, Wu J-J. Metallo-β-lactamases in clinical *Pseudomonas* isolates in Taiwan and identification of VIM-3, a novel variant of the VIM-2 enzyme. *Antimicrob Agents Chemother* 2001; **45**(8): 2224-8.
9. Hornsey M, Phee L, Wareham DW. A novel variant, NDM-5, of the New Delhi metallo-β-lactamase in a multidrug-resistant *Escherichia coli* ST648 isolate recovered from a patient in the United Kingdom. *Antimicrob Agents Chemother* 2011; **55**(12): 5952-4.
10. Perez-Perez FJ, Hanson ND. Detection of plasmid-mediated AmpC beta-lactamase genes in clinical isolates by using multiplex PCR. *J Clin Microbiol* 2002; **40**(6): 2153-62.
11. Woodford N, Ellington MJ, Coelho JM, Turton JF, Ward ME, Brown S, Amyes SG, Livermore DM. Multiplex PCR for genes encoding prevalent OXA carbapenemases in *Acinetobacter* spp. *Int J Antimicrob Agents* 2006; **27**(4): 351-3.
12. Lam MM, Seemann T, Tobias NJ, Chen H, Haring V, Moore RJ, Ballard S, Grayson LM, Johnson PD, Howden BP. Comparative analysis of the complete genome of an epidemic hospital sequence type 203 clone of vancomycin-resistant *Enterococcus faecium*. *BMC Genomics* 2013; **14**(1): 595.
