# Supplementary material for: An ISO-certified genomics workflow for identification and surveillance of antimicrobial resistance
Source: Nat Commun. 2023 Jan 4;14:60. doi: 10.1038/s41467-022-35713-4 (PMC9813266; doi:10.1038/s41467-022-35713-4)
Supplement: Supplementary file 3 — Description of Additional Supplementary Files [file 41467_2022_35713_MOESM3_ESM.pdf]

Description of Additional Supplementary Files:

Supplementary Data 1: Example outputs from abritAMR tool

Supplementary Data 2: Sequence data for validation datasets
