# Supplementary material for: An ISO-certified genomics workflow for identification and surveillance of antimicrobial resistance
Source: Nat Commun. 2023 Jan 4;14:60. doi: 10.1038/s41467-022-35713-4 (PMC9813266; doi:10.1038/s41467-022-35713-4)
Supplement: Supplementary file 6 — Reporting Summary [file 41467_2022_35713_MOESM6_ESM.pdf]

nature portfolio

Corresponding author(s): Benjamin Howden
Last updated by author(s): Dec 9, 2022

Reporting Summary

Nature Portfolio wishes to improve the reproducibility of the work that we publish. This form provides structure for consistency and transparency in reporting. For further information on Nature Portfolio policies, see our Editorial Policies and the Editorial Policy Checklist.

Statistics

For all statistical analyses, confirm that the following items are present in the figure legend, table legend, main text, or Methods section.

n/a	Confirmed	
x	☐	The exact sample size (n) for each experimental group/condition, given as a discrete number and unit of measurement
x	☐	A statement on whether measurements were taken from distinct samples or whether the same sample was measured repeatedly
x	☐	The statistical test(s) used AND whether they are one- or two-sided<br>*Only common tests should be described solely by name; describe more complex techniques in the Methods section.*
x	☐	A description of all covariates tested
x	☐	A description of any assumptions or corrections, such as tests of normality and adjustment for multiple comparisons
☐	x	A full description of the statistical parameters including central tendency (e.g. means) or other basic estimates (e.g. regression coefficient) AND variation (e.g. standard deviation) or associated estimates of uncertainty (e.g. confidence intervals)
x	☐	For null hypothesis testing, the test statistic (e.g. F, t, r) with confidence intervals, effect sizes, degrees of freedom and P value noted<br>*Give P values as exact values whenever suitable.*
x	☐	For Bayesian analysis, information on the choice of priors and Markov chain Monte Carlo settings
x	☐	For hierarchical and complex designs, identification of the appropriate level for tests and full reporting of outcomes
x	☐	Estimates of effect sizes (e.g. Cohen's d, Pearson's r), indicating how they were calculated

Our web collection on statistics for biologists contains articles on many of the points above.

Software and code

Policy information about availability of computer code

Data collection	No software used
Data analysis	abritAMR pipeline v1.0.3 - custom code, GitHub link: https://github.com/MDU-PHL/abritAMR<br>Other code used is from open source software, and links to publications given in manuscript:<br>Shovill v1.1.0 (https://github.com/kristyhoran/shovill-1)<br>AMRFinderPlus tool v3.10.21 (https://github.com/ncbi/amr) and database (https://github.com/ncbi/amr/wiki/AMRFinderPlus-database)<br>SPAdes v3.15.4 (https://github.com/ablab/spades)<br>SKESA v2.4.0 (https://github.com/ncbi/SKESA/releases)<br>epiR package (https://www.rdocumentation.org/packages/epiR/versions/2.0.53) - in statistical analysis section<br>Used with RStudio version 1.4.1717.

For manuscripts utilizing custom algorithms or software that are central to the research but not yet described in published literature, software must be made available to editors and reviewers. We strongly encourage code deposition in a community repository (e.g. GitHub). See the Nature Portfolio guidelines for submitting code & software for further information.

March 2021

Data

Policy information about availability of data

All manuscripts must include a data availability statement. This statement should provide the following information, where applicable:

- Accession codes, unique identifiers, or web links for publicly available datasets
- A description of any restrictions on data availability
- For clinical datasets or third party data, please ensure that the statement adheres to our policy

Data availability. Accession numbers for genomes used in validation and generation of synthetic reads in this study, including PCR results for validation dataset, are supplied in Supplementary Data 2 and uploaded to NCBI Sequence Read Archive (BioProjects PRJNA529744 (https://www.ncbi.nlm.nih.gov/bioproject/?term=PRJNA529744), PRJNA565795 (https://www.ncbi.nlm.nih.gov/bioproject/?term=PRJNA565795), PRJNA856406 (https://www.ncbi.nlm.nih.gov/bioproject/?term=PRJNA856406), PRJNA856415 (https://www.ncbi.nlm.nih.gov/bioproject/?term=PRJNA856415), PRJNA857525 (https://www.ncbi.nlm.nih.gov/bioproject/?term=PRJNA857525), PRJNA857526 (https://www.ncbi.nlm.nih.gov/bioproject/?term=PRJNA857526), PRJNA857528 (https://www.ncbi.nlm.nih.gov/bioproject/?term=PRJNA857528), PRJNA857531 (https://www.ncbi.nlm.nih.gov/bioproject/?term=PRJNA857531), PRJNA857533 (https://www.ncbi.nlm.nih.gov/bioproject/?term=PRJNA857533), PRJNA857534 (https://www.ncbi.nlm.nih.gov/bioproject/?term=PRJNA857534), PRJNA870170 (https://www.ncbi.nlm.nih.gov/bioproject/?term=PRJNA870170) and PRJNA319593 (https://www.ncbi.nlm.nih.gov/bioproject/?term=PRJNA319593)) as well as the abritAMR GitHub repository (https://github.com/MDU-PHL/abritAMR). Source data for figures are provided with this paper.

Code availability. Code for the abritAMR pipeline is publicly available at https://github.com/MDU-PHL/abritAMR.

Human research participants

Policy information about studies involving human research participants and Sex and Gender in Research.

Reporting on sex and gender	N/A
Population characteristics	N/A - based on isolates
Recruitment	N/A - based on isolates
Ethics oversight	Study is based on isolates collected as part of routine public health surveillance. As de-identified data were used for the validation study, ethics approval was not required.

Note that full information on the approval of the study protocol must also be provided in the manuscript.

Field-specific reporting

Please select the one below that is the best fit for your research. If you are not sure, read the appropriate sections before making your selection.

[x] Life sciences [ ] Behavioural & social sciences [ ] Ecological, evolutionary & environmental sciences

For a reference copy of the document with all sections, see nature.com/documents/nr-reporting-summary-flat.pdf

Life sciences study design

All studies must disclose on these points even when the disclosure is negative.

Sample size	Sample sizes based on requirements for ISO accreditation process
Data exclusions	No data exclusions
Replication	Reproducibility was measured as part of the ISO accreditation process, with a set of 13 samples replicated within sequencing runs (at least 3 times) and across sequencing runs, described in precision testing and is described as repeatability and reproducibility in results (Table 1). Bioinformatic analyses were also reproduced independently by two bioinformaticians.
Randomization	As the study design was a validation study, where all isolate sequences were tested for each applicable section, randomisation was not applicable in this study.
Blinding	Bioinformaticians were blinded to the PCR results in the PCR validation dataset whilst generating the data, until analysis.<br>With the synthetic dataset analysis, detection of AMR determinants from both synthetic data and whole genomes was performed independently, and only analysed together once complete to determine correlation.

Reporting for specific materials, systems and methods

We require information from authors about some types of materials, experimental systems and methods used in many studies. Here, indicate whether each material, system or method listed is relevant to your study. If you are not sure if a list item applies to your research, read the appropriate section before selecting a response.

Materials & experimental systems

n/a	Involved in the study	
☒	☐	Antibodies
☒	☐	Eukaryotic cell lines
☒	☐	Palaeontology and archaeology
☒	☐	Animals and other organisms
☒	☐	Clinical data
☒	☐	Dual use research of concern

Methods

n/a	Involved in the study	
☒	☐	ChIP-seq
☒	☐	Flow cytometry
☒	☐	MRI-based neuroimaging
